# Emodin protected against retinal ischemia insulted neurons through the downregulation of protein overexpression of β-catenin and vascular endothelium factor

**DOI:** 10.1186/s12906-020-03136-7

**Published:** 2020-11-10

**Authors:** Howard Wen-Haur Chao, Yu-Kuang Chen, Jorn-Hon Liu, Hwai-Tzong Pan, Hsin-May Lin, Hsiao-Ming Chao

**Affiliations:** 1 Department of Ophthalmology, Cheng Hsin General Hospital, Taipei, Taiwan; 2 Institute of Pharmacology, School of Medicine, National Yang-Ming University, Taipei, Taiwan; 3 Department of Chinese Medicine, School of Chinese Medicine, China Medical University, Taichung, Taiwan

**Keywords:** Retinal ischemia, Emodin, β-Catenin, Vascular endothelium factor

## Abstract

**Background:**

Emodin has been proved to have an anti-ischemic effect on the brain, however little research has been done on its effect on vision-threatening retinal ischemia. Thus, an investigation was carried out into the hypothetical efficacy of emodin against retinal ischemia and the role of β-catenin/VEGF in its therapeutic mechanism.

**Methods:**

Retinal ischemia, followed by reperfusion (IR), was inducted by raising the intraocular pressure of a Wistar rat’s eye to 120 mmHg for 60 min. Additionally, pre-ischemic/post-ischemic intravitreous injections of emodin (4, 10 and 20 μM) or vehicle were carried out on the eye with retinal ischemia. MTT assay, electroretinograms, cresyl violet staining retinal thickness measurements, and fluorogold retrograde labelling of retinal ganglion cells (RGCs) as well as Western blotting were carried out.

**Results:**

Cultured RGC-5 cells subjected to oxygen glucose deprivation (OGD) were used to confirm the effective concentrations of emodin (administered 1 h pre-OGD, pre-OGD emodin). The most effective and significant (*P* = 0.04) dose of pre-OGD emodin was observed at 0.5 μM (cell viability: 47.52 ± 3.99%) as compared to pre-OGD vehicle treatment group (38.30 ± 2.51%). Furthermore, pre-ischemic intravitreous injection of 20 μM emodin (Emo20 + IR = 0.99 ± 0.18, *P* < 0.001) significantly attenuated the ischemia induced reduction in ERG b-wave amplitude**,** as compared to pre-ischemic intravitreous vehicle (Vehicle+IR = 0.04 ± 0.02). Post-ischemic intravitreous 20 μM emodin also significantly (*P* < 0.001) attenuated the ischemia associated b-wave reduction (IR + Em20 = 0.24 ± 0.09). Compared with pre-ischemic intravitreous vehicle (Vehicle+IR; whole retina thickness = 71.80 ± 1.08 μm; inner retina thickness = 20.97 ± 0.85 μm; RGC =2069.12 ± 212.82/0.17mm^2^), the significant (*P* < 0.001) protective effect was also present with pre-ischemic administration of emodin. This was shown by observing cresyl violet stained retinal thickness (Emo20 + IR: whole retina = 170.10 ± 0.10 μm; inner retina = 70.65 ± 2.06 μm) and retrograde fluorogold immunolabeled RGC density (4623.53 ± 179.48/0.17mm^2^). As compared to the normal control (the ratio of β-catenin/VEGF to β-actin was set as 1 in the Sham group), the β-catenin/VEGF protein level significantly (*P* < 0.001) increased after retinal ischemia and when pre-ischemic intravitreous vehicle (Vehicle+IR = 1.64 ± 0.14/7.67 ± 2.57) was carried out. However, these elevations were significantly (*P* = 0.02) attenuated by treatment with emodin 20 μM (Emo20 + IR = 1.00 ± 0.19/1.23 ± 0.44).

**Conclusions:**

The present results suggest that emodin might protect against retinal ischemia insulted neurons such as RGCs by significantly downregulating the upregulation of β-catenin/VEGF protein that occurs during ischemia.

**Supplementary Information:**

**Supplementary information** accompanies this paper at 10.1186/s12906-020-03136-7.

## Background

### Clinical relevance of the retinal ischemic model

Retinal vascular occlusion, glaucomatous optic neuropathy, proliferative diabetic retinopathy, and neovascular age-related macular degeneration as well as retinal developmental anomalies are conditions that have been associated with retinal ischemia [1–3]. Clinically, retinal ischemia is detected when there are alterations in the b-wave of the electroretinogram (ERG), the presence of optical coherence tomography-proved retinal thinning, and/or alterations in visual field due to the death of inner retinal neurons (e.g. retinal ganglion cells, RGCs) [1]. These diseases affect millions of people worldwide, and therefore the management of retina ischemia is very important. A model involving the induction of retinal ischemia was therefore established that involved increasing intraocular pressure (IOP). Using this approach, an investigation into novel therapeutic approaches related to various signalling pathways would be a useful way to finding an appropriate agent against retinal ischemia.

### Exploring β-catenin and VEGF

Studies have shown that β-catenin signaling pathway activates T-cell factor-4 (TCF-4) and promotes cell proliferation under normoxic condition [4]. However, in hypoxia, β-catenin promotes the expression of hypoxia-inducible factor-1α (HIF-1α), which is widely known to subsequently elevate the level of vascular endothelial growth factor (VEGF) [5]. This event in turn arrests the cell cycle as an adaptation to hypoxia [6]. Additionally, the inhibition of protein expression of β-catenin and/or VEGF have been reported to prevent an ischemia induced increase in vascular permeability that is related to consequential neovascularization, ocular hemorrhage and/or cystoid macular edema) [7–9].

### The rationale for administration of emodin using the retinal ischemia animal model

The traditional Chinese Herb Dà Huáng, also commonly known as Rhubarb (*Rheum rhabarbarum*), has been used for thousands of years in China for its anti-carcinogenic, antipyretic, anti-inflammatory and antioxidative medicinal properties [8]. Emodin is one of the active ingredients of *Rheum rhabarbarum*. Emodin-8-O-beta-D-glucoside has been proved to have a neuroprotective effect against cerebral ischemia and glutamate induced neuronal damage via its antioxidative properties and its inhibition of glutamate neurotoxicity [9]. Additionally, emodin has been shown to possess antioxidative effects via its ability to prevent lipid membrane peroxidation, the scavenging of free radicals, and the inhibition of free-radical generation [10]. The aforementioned effects on the production of oxygen free radicals, lipid peroxidation and glutamate excitotoxicity have been observed in the brain [9] and it is well known that a similar retinal ischemic cascade occurs in the retina, also resulting in neuronal death [11–13]. It would thus be very interesting to see whether or not emodin would be able to alleviate retinal ischemic damage.

In one previous study, a RGC density of 2626 ± 220/mm^2^ was observed in the retinas of normal SD rats [14]. Three weeks after ischemia plus reperfusion (IR), a significant decrease in the RGC density was identified in the ischemic retina with (IR + Vehicle = 1367 ± 123) or without (only IR = 1289 ± 109) post-ischemic intravitreous injection of vehicle. Interestingly, there was a significant increase in the cell density of the ischemic retinas that had received post-ischemic emodin, a protein kinase CK2 inhibitor (IR + Emodin = 2263 ± 187). Activation of CK2 was found to increase intracellular zinc levels, which in turn lead to neuronal death [15]. The antagonism of CK2 by emodin was able to bring about the above neuroprotection of RGCs. Additionally, significantly higher levels of fibronectin have been observed in diabetic rat retinas when compared with normal control rats [16, 17] and this contrasted with the result in diabetic rats after the treatment of emodin where there was a significant reduction in the retinal levels of fibronectin [17]. The above findings indicated emodin has an inhibitory effect on the progression of diabetic retinopathy in rats.

The present investigation also explores whether emodin can ameliorate the in vitro oxygen glucose deprivation (OGD; ischemia simulation) model induced reduction in retinal cell viability. The protective effect of emodin was initially analysed using 3-(4,5-dimethylthiazol-2-yl)-2,5-diphenyltetrazolium bromide (MTT) cell viability assay. Using this cellular test, appropriate concentrations of emodin were selected for the treatment in the present animal experiments.

Taken together, the findings from the above studies support the hypothesis that emodin possesses antioxidative properties [8], is antagonistic to excitotoxicity [9], is able to protect RGCs against retinal ischemia [14] and is able to inhibit the progression of diabetic retinopathy [17]. Therefore, in the present study, an animal model was used to investigate the hypothesis that emodin has protective effect(s) against retinal ischemia in the electrophysiological, histopathological and immunohistochemical aspects. Furthermore, an investigation was made into how levels of β-catenin/VEGF in the ischemic retina might be regulated during treatment with emodin.

## Methods

### Cellular experiments

#### Oxygen glucose deprivation

RGC-5 cells have been reported to be not transformed rat RGCs, but rather are mouse retinal neuronal precursor cells [18]. OGD [19] was established by incubating RGC-5 cells in Dulbecco’s modified Eagle medium (DMEM; Thermo Fisher Scientific Inc.) without glucose at 37 °C under ischemia simulation conditions, namely 1% O_2_ (measured using a Penguin Incubator; Astec Company, Kukuoka, Japan), 94% N_2_ and 5% CO_2_. Various experimental groups (Table 1) were investigated. These were cells treated with: (i) culture medium containing vehicle (control), (ii) 1 h of pre-OGD treatment with vehicle (Pre-OGD vehicle), (iii) 1 h of pre-OGD treatment with 0.25 μM emodin (Pre-OGD Emo 0.25 μM), (iv) 1 h of pre-OGD treatment with 0.5 μM emodin (Pre-OGD Emo 0.5 μM). Following 24 h of OGD, the cell cultures were transferred to new DMEM for another 1 day. MTT assays to assess cell viability were then carried out.

**Table 1:** Quantitative analysis of the viability of the cultured RGC-5 cell line utilizing MTT assay

Group (*n* = 5 ~ 6)	Control	Pre-OGD vehicle	Pre-OGD Emo 0.25 μM	Pre-OGD Emo 0.5 μM
Cell viability (% of control)	100	38.30 ± 2.51%***	43.81 ± 3.75%	47.52 ± 3.99%†

The value in each group was the ratio of the viability of the cultured RGC-5 cells relative to that of the control group, which was set as 100%. This study include 4 groups including control (normal; RGC-5 cells that were incubated in culture medium containing vehicle), OGD (1 h of pre-OGD administration of vehicle before the OGD condition), Pre-OGD 0.25 μM (1 h pre-OGD administration of 0.25 μM emodin before the OGD condition), and Pre-OGD 0.5 μM (1 h pre-OGD administration of 0.5 μM Emodin). *** denotes an extremely significant difference (*P* < 0.001) between the normal control and the OGD group. † denotes a significant difference (*P* = 0.04) between the OGD group and the pre-OGD application of emodin 0.5 μM group. The results are mean ± SE (*n* = 5 ~ 6). *Abbreviations*: *RGC-5* retinal ganglion cell-5, *MTT* 3-(4,5-dimethylthiazol-2-yl)-2,5-diphenyltetrazolium bromide, *OGD* oxygen glucose deprivation, *Emo* Emodin

#### MTT assay

Mitochondrial nicotinamide adenine dinucleotide phosphate dependent oxidoreductases are able to reduce MTT to produce formazan [19] and thus increased levels of dark purple formazan that are associated with greater cell viability. MTT (0.5 mg/mL; Sigma-Aldrich) was added to 100 μL of cells in each well of 96-well plates for 3 h at 37 °C. After reduction of the MTT, the formazan was dissolved by the addition of 100 μL of DMSO. After shaking, the optical density (OD) of the dissolved formazan was measured using an ELISA plate reader (Synergy H1 Multi-Mode Reader BioTek Instruments) at 562 nm. Cell viability was calculated as the change in OD value compared to the untreated control (100%).

### Animal experiments

#### Animal use

Animal Use Approval was agreed by the Institutional Animal Care and Use Committee at Cheng Hsin General Hospital, Taipei, Taiwan (Approval No: CHIACUC 107–03; **Supplementary file** **1**). The animals were Wistar rats purchased from BioLasco, Taipei. Upon arrival, the Wistar rats were 6 weeks old and weighed 175 ~ 250 g. They were raised in groups of less than six rats inside a large plastic cage (Shineteh Instruments Co., Ltd., Taipei). The humidity and temperature were controlled to be within the ranges 40 to 60% and 21 ± 2 °C, respectively. Rats were assigned randomly to the various experimental groups. The number of the rats used in this study is 120 (=99 + 21; 99 = 60 + 16 + 23; Please also **see Supplementary file** **2**). These include animals (*n* = 21) that died during the procedures; these occurred during retinal ischemic induction (*n* = 9), ERG detection (*n* = 4), and fluorogold retrograde RGC labelling (*n* = 8). After ERG recordings had been performed, all surviving rats (*n* = 99) underwent the following procedures, namely cresyl violet staining, fluorogold labelling and Western blot assays. The surviving animals, each of which had been subjected to a sham procedure, IR with pre-administered vehicle, or IR with pre−/post-administered emodin (Emo) formed the following groups: Sham (*n* = 19), Vehicle+IR (*n* = 20), Emo4 + IR (*n* = 10), Emo10 + IR (*n* = 20), Emo20 + IR (*n* = 20) and IR + Emo20 (*n* = 10). The animal experimental procedures were performed in a way that adhered to the Animal Research: Reporting of In Vivo Experiments (ARRIVE) guidelines (**Supplementary file** **3**).

#### Animal anesthesia and euthanasia

The Wistar rats were anesthetized using a combination of intraperitoneal ketamine (Pfizer; 100 mg/kg) and xylazine (Sigma-Aldrich; 5 mg/kg) to bring about pain relief and sedation [1–3]. ERG recordings, fluorogold intracranial injections and intravitreous injections of defined agents were performed. To minimise pain and to humanely sacrifice the Wistar rats, a final intraperitoneal injection of at least 140 mg/kg of sodium pentobarbital (SCI Pharmtech) was given to each subject (Scientific Procedures Acts 1986) [1–3].

#### Administration of drug

Emodin was purchased from Sigma-Aldrich (E7881; 90%; storage at 2–8 °C; extracted from Frangula bark; St. Louis, Missouri, United States). Drug administration for different procedures, namely ERG, fluorogold RGC labelling, cresyl violet retinal staining and Western blot assays, are described in Tables 2, 3, 4 and 5. Vehicle or emodin (5 μL) was administered via an intravitreous injection route. A 10 μL Hamilton microsyringe connected with a 30-gauge needle was utilised for the intravitreous injections. Excess solution (> 5 μL per administration) was prepared in order to prime the needle/syringe knob of any dead volume. The rationale for an intravitreous administration route is that these agents are then able to be directly diffused to the target. Emodin was dissolved in vehicle (DMSO:distilled water = 1:3). The defined final cellular concentrations of administered drug were 0.25 or 0.5 μM, which were achieved after 40 times dilution from the stock concentrations, namely 10 or 20 μM, based on an arbitrary definition of the vitreous volume as 200 μL. The defined diluted concentration of the solvent is non-toxic as described [20]. To test a wider range of dose-response, 4 μM of emodin was thus added in the animal experiments. The Vehicle+IR and Emo + IR groups (pre-ischemic administration of emodin 4, 10 or 20 μM) were subjected to the following experimental procedures; these were initial pre-ischemic intravitreous injection (vehicle or emodin), retinal ischemia induction 1 day later, and sacrifice of the animal on the following day to allow the various post-mortem procedures except ERG. In the post-ischemic treatment group, in order to decrease the number of animals used, the present study only used a single emodin concentration of 20 μM. Furthermore, intravitreous emodin was given 1 day after initial retinal ischemia induction and animals were sacrificed the following day to allow the performance of various post-mortem procedures as mentioned above.

**Table 2:** Group names and conditions used at various groups in the ERG b-wave analysis

Group name	Definition of conditions in which the retina has been treated
Sham (*n* = 7; control)	Sham procedure
Vehicle+IR (*n* = 8)	Pre-ischemic intravitreous injection of vehicle followed by IR
Emo4 + IR (*n* = 8)	Pre-ischemic intravitreous injection of 4 μM emodin followed by IR
Emo10 + IR (*n* = 4)	Pre-ischemic intravitreous injection of 10 μM emodin followed by IR
Emo20 + IR (*n* = 4)	Pre-ischemic intravitreous injection of 20 μM emodin followed by IR
IR + Emo20 (*n* = 9)	IR followed by post-ischemic intravitreous injection of 20 μM emodin

*Abbreviations*: *ERG* electroretinogram, *IR* ischemia/reperfusion

**Table 3:** Group names and conditions at various groups evaluating the cresyl violet stained retinal thickness

Group name	Definition of conditions in which the retina has been treated
Sham (*n* = 10; control)	Sham procedure
Vehicle+IR (*n* = 10)	Pre-ischemic intravitreous injection of vehicle followed by IR
Emo4 + IR (*n* = 10)	Pre-ischemic intravitreous injection of 4 μM emodin followed by IR
Emo10 + IR (*n* = 10)	Pre-ischemic intravitreous injection of 10 μM emodin followed by IR
Emo20 + IR (*n* = 10)	Pre-ischemic intravitreous injection of 20 μM emodin followed by IR
IR + Emo20 (*n* = 10)	IR followed by post-ischemic intravitreous injection of 20 μM emodin

*Abbreviation*: *IR* ischemia/reperfusion

**Table 4:** Group names and conditions at various groups analysing retrograde fluorogold labelled RGCs

Group name	Definition of conditions in which the retina has been treated
Sham (*n* = 4; control)	Sham procedure
Vehicle+IR (*n* = 4)	Pre-ischemic intravitreous injection of vehicle followed by IR
Emo10 + IR (*n* = 4)	Pre-ischemic intravitreous injection of 10 μM emodin followed by IR
Emo20 + IR (*n* = 4)	Pre-ischemic intravitreous injection of 20 μM emodin followed by IR

*Abbreviations*: *RGCs* retinal ganglion cells, *IR* ischemia/reperfusion

**Table 5:** Group names and conditions at various groups in the western blotting analysis of β-catenin/VEGF protein

Group name	Definition of conditions in which the retina has been treated
Sham (*n* = 5; control)	Sham procedure
Vehicle+IR (*n* = 6)	Pre-ischemic intravitreous injection of vehicle followed by IR
Emo10 + IR (*n* = 6)	Pre-ischemic intravitreous injection of 10 μM emodin followed by IR
Emo20 + IR (*n* = 6)	Pre-ischemic intravitreous injection of 20 μM emodin followed by IR

*Abbreviations*: *VEGF* vascular endothelium growth factor, *IR* ischemia/reperfusion

#### Induction of retinal ischemia

The procedure used raised the IOP and was designed to cause retinal ischemia in Wistar rats [1–3]. The rats were anesthetized and constrained with a sterotaxic frame. A 30-guage needle connected to an elevated saline bottle was attached to anterior chamber of the rat’s eye. The pressure exerted by the saline inside the bottle was controlled to 120 mmHg. This procedure was conducted for 1 h. Successful retinal ischemia induction was confirmed by observing that whitening of the retina took place [1–3]. Throughout the experiments, the animals were kept on heat mats to maintain their body temperature. The sham procedure involved keeping the pressure in the saline bottle 0 mmHg [3].

#### ERG recording

Animals were given anaesthesia prior to the flash ERG measurements. For the Sham or pre-ischemic treatment group (intravitreous injection of emodin or vehicle 1 day before ischemia; Emodin+IR or Vehicle+IR), flash ERG responses were measured before the sham procedure, IR procedures or any drug administration (day 0), and one day after the sham procedure or IR procedure with pre-ischemic intravitreous injection. In the post-ischemic treatment group, ERG recordings were collected pre-ischemia (day 0), and post-ischemia (at timepoints: 1 day after ischemia; 1 day after post-ischemic intravitreous injection of emodin). Dark adaption and pupil dilation using 1% tropicamide and 2.5% phenylephrine were carried out 8 h before ERG measurement. The ERG recording machinery was purchased from the Grass-Telefactor Company (AstroNova, QC, Canada) and included a stimulator (PS22), a regulated power supply (RPS107), and an amplifier (P511). A strobe light (0.5 Hz), which acted as the source of stimulus, was placed 2 cm directly in front of the rat’s eye. Fifteen consecutive measurements (10 kHz) were retrieved every two seconds. The amplitudes were calculated to obtain an average. To make comparisons between the various groups, the ratios of the b-wave amplitude of one eye (sham procedure or ischemic insult with defined agents) to that of the untreated fellow normal eye were analysed.

#### Cresyl violet staining

After the aforementioned sacrifice of rats, the animals underwent intracardial perfusion with normal saline. The eyeballs were then enucleated, fixed with 4% paraformaldehyde for 1 day at 4 °C, dehydrated with ethanol, embedded in paraffin (Tissue-Tek TEC 5; Sakura) and sectioned into 5 μm thickness slices. The sectioned samples were then stained with cresyl violet and examined using a light microscope (Leica). All sectioned retinal samples were photographed under the same magnification (Ilford Pan-F plus film, 50 ASA).

The thickness of a retina was determined by sectioning retinal samples at the same distance (1.5 mm from the disc). To evaluate the degree of the injury caused by retinal ischemia, the whole retinal thickness [from the inner limiting membrane (ILM) to the retinal pigment epithelium (RPE) layer] and the inner retinal thickness [from the ILM to the inner nuclear layer (INL)] were measured. All the measurements were carried out by an expert who was unaware of the experimental conditions used to treat the samples.

#### Retrograde fluorogold labeling of RGCs

On the initial day, after receiving anesthesia, a 2-cm opening was made in the skin of the rat’s head and two round side openings in the skull were created by a drill. These were followed by an intracranial injection of 2 μL 0.5% fluorogold (Sigma-Aldrich) through a Hamilton microsyringe at depths of 3.8, 4.0, and 4.2 mm below the level of the skull vertex. One day following the fluorogold treatment, either the pre-ischemic emodin, vehicle or sham procedure were carried out (as described in the section administration of drug). The rats were sacrificed 3 days after the fluorogold injection was performed. After sacrifice, the retinal tissue was carefully isolated, incubated with 4% paraformaldehyde fixative, sectioned and processed. Finally, the samples were then analysed by fluorescent microscopy. The RGC density was defined as the total RGC amount divided by the whole retinal area and its mean was then calculated.

#### Western blotting

Samples of the retina were retrieved immediately after the sacrifice of the rats. Denatured proteins were then obtained using lysis buffer (mammalian protein extraction reagent; Hycell), the samples were then sonicated and quantified to give equivalent amounts of 30 μg/30 μl/well. The prepared protein samples were separated by 12% sodium dodecyl sulfate polyacrylamide gel electrophoresis (Bio-Rad, Hercules, CA) and then transferred to a polyvinylidene fluoride (PVDF) membrane. The PVDF membranes were incubated with blocking buffer (135 mM NaCl, 8.1 mM Na_2_HPO_4_, 1.5 mM KH_2_PO_4_, 2.7 mM KCl; pH 7.2) containing 5% fat-free skimmed milk at 4 °C for 16 h. Next, the membranes were incubated with various primary antibodies at 25 °C for 1 h, there were mouse anti-β-actin monoclonal antibody (ab6276; 1:5000; Abcam Inc., Cambridge, UK), rabbit anti-β-catenin monoclonal antibody (ab32572; 1:5000; Abcam Inc., Cambridge, UK) and rabbit polyclonal anti-VEGF antibody (A-20; 1:200; sc-152). The blots were subsequently incubated with their appropriate secondary antibody, either horseradish peroxidase-conjugated goat anti-rabbit IgG (111–035-003; 1:2000; Jackson ImmunoResearch) or horseradish peroxidase-conjugated goat anti-mouse IgG (sc-2005; 1:2000; Santa Cruz Biotech, Santa Cruz, CA) at 25 °C for 1 h. Finally, the membranes were then developed using an enhanced chemiluminescent analysis system (HyCell) and scanned using an imager (Amersham Imager; LifeSciences); the amount of each protein was quantified according to scanning densitometry.

#### Analysis of statistical significance

Unpaired Student *t*-tests were used to compare the statistical difference between two experimental groups. Probability *(P)* of *<* 0.05 was taken to represent a statistical significant difference. All of the results are shown as mean ± standard error.

## Results

### In vitro

#### Effect of emodin on OGD-induced decrease in cell viability as evaluated by MTT assay

**Fig. 1:**
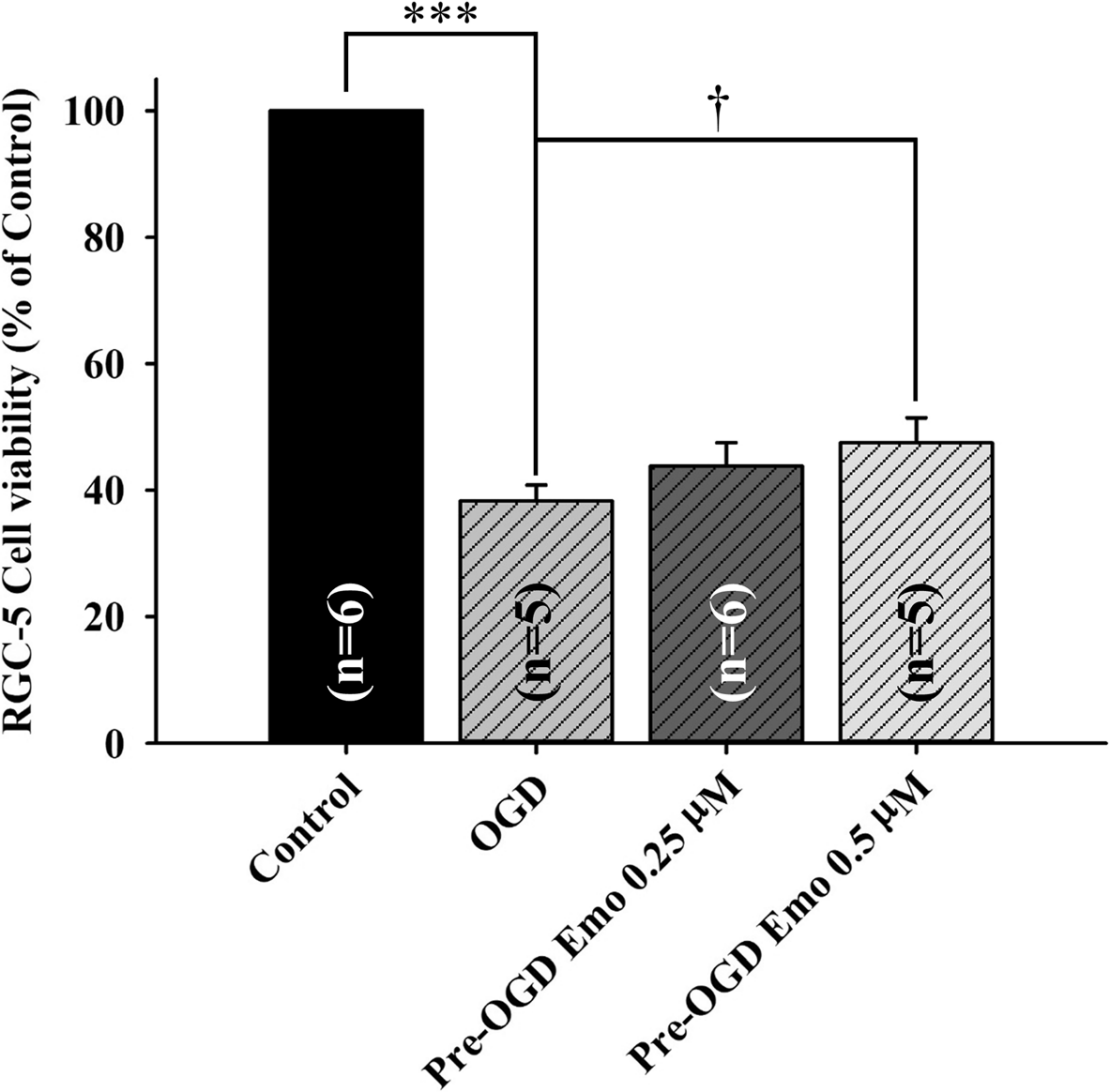
The viability of the cultured RGC-5 cell line was quantitatively analysed using MTT assay. The value in each group was the ratio of the viability of the cultured RGC-5 cells relative to that of the control group, which was set as 100%. This study includes four groups, namely control (normal; RGC-5 cells which were cultured in culture medium containing vehicle), OGD (1 h of pre-OGD treatment with vehicle), Pre-OGD 0.25 μM (1 h of pre-OGD treatment with 0.25 μM emodin), and Pre-OGD 0.5 μM (1 h of pre-OGD treatment with 0.5 μM Emodin). *** identifies that the normal control significantly differs (*P* < 0.001) from the OGD group. † designates a significant difference (*p* = 0.04) between the OGD group and the pre-OGD application of emodin 0.5 μM group. The results are mean ± SE (*n* = 5 ~ 6). Abbreviations: RGC-5, retinal ganglion cell-5; MTT, 3-(4,5-dimethylthiazol-2-yl)-2,5-diphenyltetrazolium bromide; OGD, oxygen glucose deprivation; Emo, Emodin

The cultured cells were used to identify the effective dose(s) of the tested agent emodin. The effect of emodin (0.25 and 0.5 μM) on the cultured cells was evaluated utilising MTT cell viability assays and a cellular ischemia simulation model, namely OGD, for 1 day. The definition of OGD [19] is that the cells were grown in culture medium without glucose at 37 °C under hypoxic conditions, namely 1% O_2_. The results for the various experimental groups versus the control (set as 100%; cells incubated in culture medium containing vehicle) are described subsequently (Fig. 1 and Table 1). Cells were incubated in culture medium plus one hour of pre-OGD administration of: vehicle (cell viability of 38.30 ± 2.51%), emodin 0.25 μM (cell viability of 43.81 ± 3.75%) and emodin 0.5 μM (cell viability of 47.52 ± 3.99%). As compared to the vehicle treatment group, emodin treatment resulted in a dosage related and significant (*P* = 0.04; at 0.5 μM) attenuation of the OGD induced cellular injury.

### In vivo

#### Effect of emodin on ischemia-induced reduced ERG b-wave amplitude

**Fig. 2:**
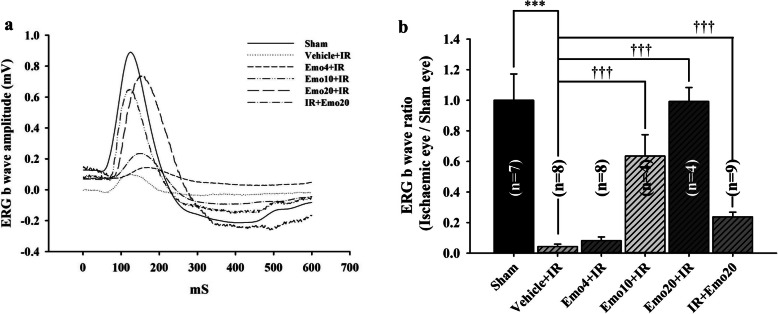
Summary of ERG b-wave measurement. **a**. Electroretinogram (ERG) b-wave amplitude. After ischemia/reperfusion (IR), a substantially reduced ERG b-wave amplitude was demonstrated in the ischemic retina pre-administered with intravitreous vehicle (Vehicle+IR) as compared with the control retina (Sham). In a dose-dependent way, pre-administered intravitreous emodin [4, 10 and 20 μM, i.e. Emo4 + IR, Emo10 + IR and Emo20 + IR], but not vehicle, ameliorated the ischemia induced reduction in ERG b-wave amplitude. Post-ischemic intravitreous injection of 20 μM emodin (IR + Emo20) also had an anti-ischemic effect. **b**. The analysis of ERG b-wave ratios revealing the efficacy of pre-ischemic and post-ischemic administration of emodin on the ischemic retinas. As compared to the ERG b-wave ratio of the Control (Sham = 0.82 ± 0.14: normalized to 1; *n* = 7), a significant (***; *P* < 0.001) reduction in that of the Vehicle+IR group was revealed. In contrast to the Vehicle+IR group (0.04 ± 0.01; *n* = 8), pre-ischemic intravitreous injection of emodin dose-responsively (Emo4 + IR = 0.08 ± 0.02, *n* = 8; Emo10 + IR = 0.64 ± 0.14, *n* = 4; then, Emo20 + IR = 0.99 ± 0.09, *n* = 4) and significantly (†††; *P* < 0.001; Emo10 + IR; Emo20 + IR) attenuated ischemic insult. Post-ischemic intravitreous injection of 20 μM emodin (IR + Emo20 = 0.24 ± 0.03; *n* = 9) had a significant (†††; *P* < 0.001) anti-ischemic effect, too. Data are mean ± S.E.M. of the number of animals shown in the parenthesis. Abbreviations for group names are provided in Table 2

In the Sham group, the ERG b-wave amplitude/ratio was measured to be 0.87 mV/1.00 ± 0.00 (Fig. 2a; amplitude ratio of 0.82 ± 0.14 normalized to 1 in Fig. 2b; Table 2; *n* = 7). Following retinal ischemia, an extremely significant drop (*P* < 0.001) in ERG b-wave amplitude/ratio was observed (Fig. 2a/b; Vehicle+IR = 0.03 mV/0.04 ± 0.02; *n* = 8). Pre-ischemic intravitreous injection of emodin alleviated the ischemia induced significant decrease in ERG b-wave amplitude/ratio (Fig. 2a/b; Emo 4.0 μM + IR = 0.07 mV/0.08 ± 0.07, *n* = 8; Emo 10.0 μM + IR = 0.58 mV/0.64 ± 0.28, *P* < 0.001, *n* = 4; Emo 20.0 μM + IR = 0.63 mV/0.99 ± 0.18, *P* < 0.001, *n* = 4). Interestingly, it was also demonstrated that post-ischemic intravitreous emodin also significantly (*P* < 0.001) ameliorated the above ischemia induced reduction in ERG b-wave amplitude/ratio (Fig. 2a/2; IR + Emo 20 μM = 0.12 mV/0.24 ± 0.09, *n* = 9).

#### Effect of emodin on ischemia-induced reduction in cresyl violet stained retinal thickness

**Fig. 3:**
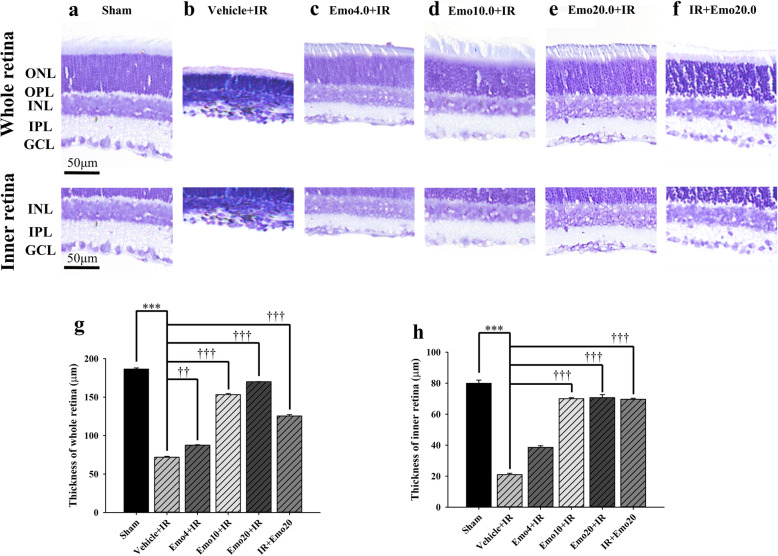
Calculation of thickness of cresyl violet stained retinas. **a ~** **f**. Cresyl violet stained retinal sections with the same eccentricity. Micrographs showing the whole (top row) or inner retina (bottom row) thickness (μm) of different groups. **a**, **b**, **g** and **h**. As compared with the retinal thickness of the control group (Sham: 186.50 ± 1.43; inner = 79.90 ± 2.06), a substantial reduction in the whole or inner retina thickness was observed in that of the Vehicle+IR group (whole = 71.80 ± 1.08; inner = 20.97 ± 0.85). **c ~ e**, **g** and **h**. Pre-ischemic intravitreous injection of emodin dose-responsively (least effect at 4 μM; Emo4 + IR: whole = 87.40 ± 0.60, inner = 38.60 ± 1.01; then, 10 μM; greatest effect at 20 μM) and in a significant way (Emo10 + IR: whole = 153.20 ± 1.48, inner = 70.05 ± 0.60; Emo20 + IR: whole: 170.10 ± 0.10; 70.65 ± 2.06) attenuated ischemia induced reduction in the whole and inner retina thickness. f, g and h. Post-ischemic intravitreous injection of 20 μM emodin also significantly alleviated ischemia reduced whole and inner retina thickness (IR + Emo20: whole = 125.45 ± 1.68, inner = 69.65 ± 0.68). **g** and **h**. Quantitative analysis of the whole or inner retina thickness. *** or †††/†† indicates a significant (*P* < 0.001 or *P* < 0.001/*P* < 0.01) difference from the Sham or Vehicle+IR group. Abbreviations: IR, ischemia plus reperfusion; ONL, outer nuclear layer; OPL, outer plexiform layer; INL, inner nuclear layer; IPL, inner plexiform layer; GCL, ganglion cell layer. Scale bar = 50 μm. The results are mean ± standard error. Abbreviations for group names are provided in Table 3

As revealed in the Fig. 3 and Table 3, in the sham group (*n* = 10), the retinal thickness (μm) was measured to to be: whole retina (186.50 ± 1.43) and inner retina (79.90 ± 2.06). After induction of retinal ischemia, a significant loss of both whole retinal thickness and inner retinal thickness (Vehicle+IR; whole = 71.80 ± 1.08; inner = 20.97 ± 0.85; *P* < 0.001) was observed. However, pre-ischemic intravitreous injection of emodin (4, 10 or 20 μM) dose-dependently (n = 10; Emo4 + IR: whole = 87.40 ± 0.60, inner = 38.60 ± 1.01; Emo10 + IR: whole = 153.20 ± 1.48, inner = 70.05 ± 0.60; Emo20 + IR: whole = 170.10 ± 0.10; inner = 70.65 ± 2.06) and significantly (*P* < 0.01 at 4 μM; *P* < 0.001 at 10 or 20 μM) alleviated the retinal thickness reduction caused by retinal ischemia and reperfusion. In addition, the post-ischemic intravitreous emodin (*n* = 10; 20 μM; IR + Emo20: whole = 125.45 ± 1.68, inner = 69.65 ± 0.68) also had a significant (*P* < 0.001) anti-ischemic effectin terms of retinal thickness.

#### Effect of emodin on the ischemia-induced decrease in retrograde fluorogold labeled RGC density

**Fig. 4:**
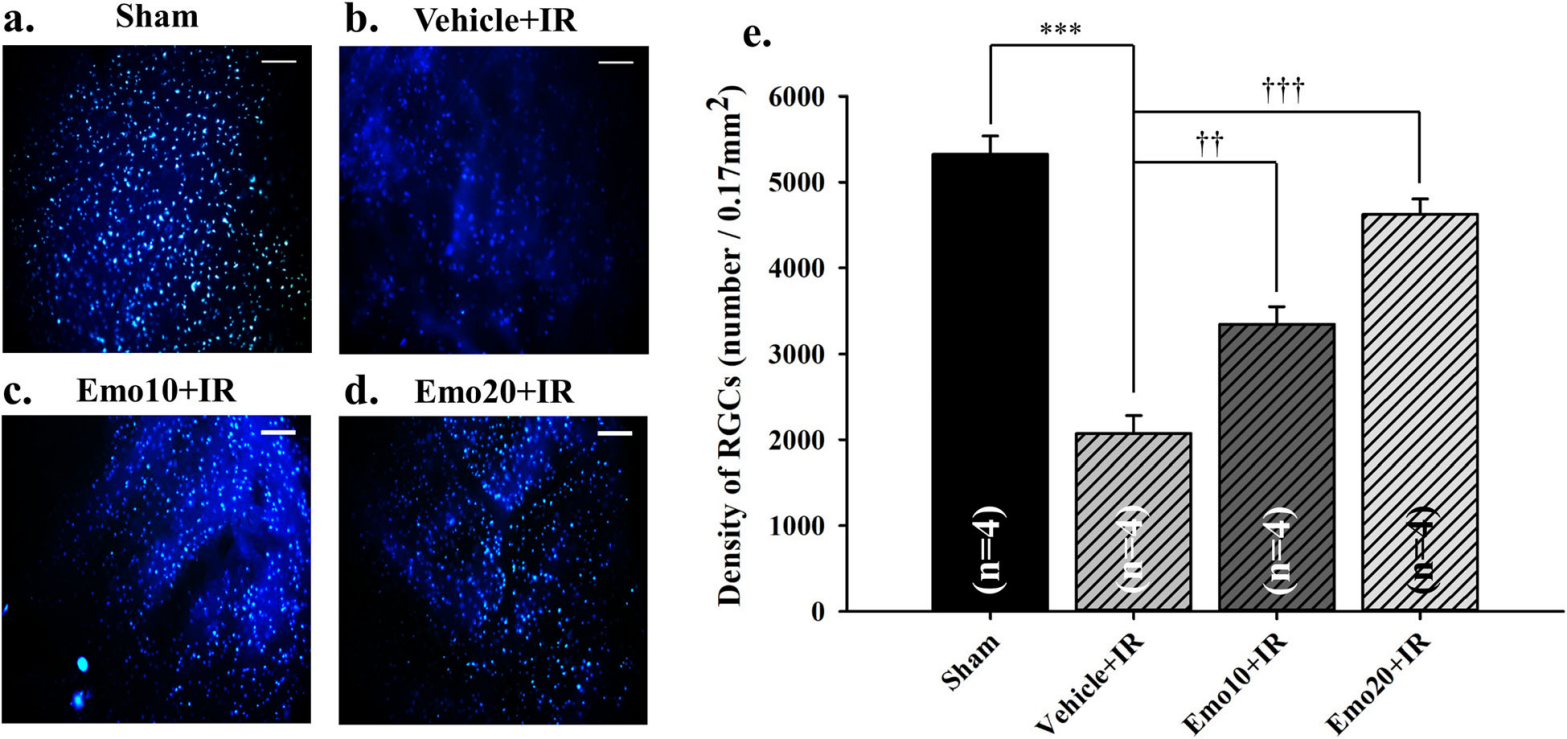
Fluorogold retrograde labelling. Micrographs showing the density of retinal ganglion cell (RGC) at various groups. a and e. At the Sham group, the highest density of RGCs was observed 5323.53 ± 215.6/0.17 mm^2^. b and e. At the Vehicle+IR group, an obvious reduction in the cell density was demonstrated. c ~ d and e. Pre-ischemic intravitreous injection of emodin 10 and 20 μM (Emo10 + IR; Emo20 + IR) in a dose dependent manner and significantly increased the cell density; e. Quantitative analysis of the RGC density. *** or ††/††† denotes a significant (*P* < 0.001 or *P* < 0.01/*P* < 0.001) difference from the Sham or Vehicle+IR group. Emodin dose-dependently and significantly counteracted the ischemia induced reduction in the RGC density. The results are mean ± standard error of the amount of animals demonstrated in the parenthesis. Scale bar is 50 μm. Abbreviation: RGC, retinal ganglion cell. Abbreviations for group names are provided in Table 4

As shown in Fig. 4a/e and Table 4, RGC immunolabeling gave a cell density of 5323.53 ± 215.6/0.17mm^2^ following the sham procedure (Sham; *n* = 4). An extremely significant drop (*P* < 0.001) in RGC density was observed in the ischemic retina with pre-ischemic intravitreous injection of vehicle (*n* = 4; Vehicle+IR = 2069.12 ± 212.82; Fig. 4b/e). Furthermore, there was a dose-dependent and significant (*n* = 4; *P* < 0.01 at 10 μM, Emo10 + IR = 3345.59 ± 206.80, Fig. 4c/e; *P* < 0.001 at 20 μM, Emo20 + IR = 4623.53 ± 179.48, Fig. 4d/e) increase in the cell density after pre-ischemic administration of either 10 or 20 μM emodin.

#### The effect of emodin on the ischemia-induced changes in the protein level ratio of β-catenin/VEGF to β-actin

**Fig. 5:**
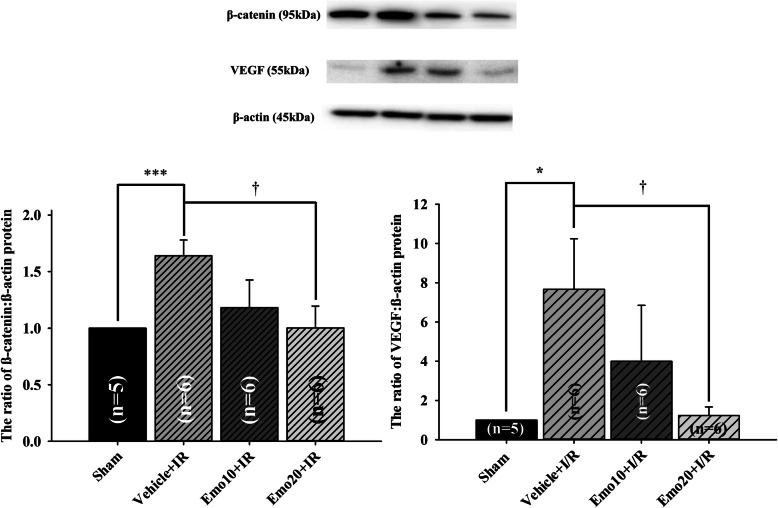
Western blot assay. Upper: blotting images of β-catenin, vascular endothelium growth factor (VEGF) and β-actin protein. Lane 1 is from a sham retina (Control); Lane 2 is the vehicle-pretreated ischemic retina (Vehicle+IR); Lanes 3 and 4 are from retinas that were subjected to IR and were pretreated with 10 μM (Emo10 + IR) and 20 μM emodin (Emo20 + IR). Lower: the bar chart analyzing the ratio of β-catenin/VEGF to the house-keeping protein β-actin. The ratio of the Sham group was normalized to 1. ***/* represents an extremely significant (*P* < 0.001) or a significant (*P* < 0.05) difference as compared to the Sham group. † represents a significant (*P* = 0.02/0.03) difference when compared to the Vehicle+IR group. As compared to the sham group, a significant increase in β-catenin/VEGF protein levels was observed after an ischemic insult and pre-ischemic application of vehicle (Vehicle+IR = 1.64 ± 0.14/7.67 ± 2.57). In contrast, pre-ischemic application of emodin dose-dependently and significantly (*P* = 0.02/0.03 at 20 μM; Emo20 + IR = 1.00 ± 0.19/1.23 ± 0.44) inhibited ischemia induced increase in β-catenin/VEGF protein levels. The values are mean ± S.E of the number of animals illustrated in the parenthesis. Abbreviations are listed as follows. IR: ischemia plus reperfusion; Pre-ischemic emodin 10 μM followed by IR (Emo10 + IR); Pre-ischemic emodin 20 μM followed by IR (Emo20 + IR). Abbreviations for group names are provided in Table 5

Western blot assays were utilized to investigate the therapeutic mechanisms by which emodin ameliorated ischemic damage. As revealed by the immunoblotting images (Fig. 5, top; Table 5), pre-ischemic administration of 10 or 20 μM emodin (Emo10 + IR or Emo20 + IR) had a dose-dependent inhibitory effect on β-catenin/VEGF protein over-expression that was induced by ischemia compared to pre-ischemic intravitreous vehicle (Vehicle+IR). In the quantitative analysis (Fig. 5, bottom; Table 5), the effects of emodin on β-catenin/VEGF protein levels were calculated using the ratio of β-catenin/VEGF:β-actin with the Sham group (*n* = 5) normalized to 1. After an ischemic insult, it was observed that there was a dose-dependent (less effect at 10 μM; Emo10 + IR = 1.18 ± 0.24/3.99 ± 2.86; *n* = 6) and significant effect (*P* = 0.02/0.03 at 20 μM; Emo20 + IR = 1.00 ± 0.19/1.23 ± 0.44; *n* = 6) of emodin whereby it blunted the significant (*P* < 0.001) upregulation of β-catenin/VEGF protein that was brought about by ischemia compared to pre-ischemic intravitreous vehicle (Vehicle+IR = 1.64 ± 0.14/7.67 ± 2.57; *n* = 6). Results that were presented in brackets follow the format of β-catenin/VEGF.

## Discussion

### The hypothetical anti-ischemic mechanism of emodin by which there is inhibition of the ischemia induced overexpression of β-catenin and VEGF

β-Catenin upregulates the expression of HIF-1α regulated genes such as VEGF (Fig. 5) in response to ischemia [6]. This then arrests the cell cycle as an adaptive response to hypoxia [6]. Consistent with the present results, retinal ischemia and pre-ischemic administration of vehicle resulted in reduced cell viability (Fig. 1), decreased ERG b-wave amplitude (Fig. 2), a reduction in the thickness of whole and inner retinas (Fig. 3) as well as a decrease in the RGC density (Fig. 4). Following retinal ischemia, pre-ischemic emodin, but not vehicle, dose-dependently (less effect at: 0.25 μM in vitro; 4 and/or 10 μM in vivo) and significantly (0.5 μM in vitro; 20 μM in vivo) increased retinal cell viability, ERG b-wave amplitude, inner/whole retinal thickness and RGC density.

The involvement of the Wnt/β-catenin pathway has been reported to be related to the protective effect of galangin during brain ischemic injury [21], as well as it being associated with retinal Müller (glial) cell amelioration of ischemia induced neovascularization and vascular leakage [7]. Moreover, emodin has been shown to provide a neuroprotective effect against brain ischemia in rats by downregulating expression of AQP4 and Cx43 following IR [22]. The present investigation of the anti-ischemic effect of emodin on the β-catenin/VEGF signalling pathway in the retina (Fig. 5), where it has been shown to protect inner retinal neurons (Fig. 3), specifically RGCs (Fig. 4), is potentially a pioneering step forward in protecting neurons (RGCs) other than glials (Müllers) that are damaged by ischemia. Under ischemic conditions (hypoxia), upregulated HIF-1α protein competes with TCF-4 to bind with cellular β-catenin. Once bound to HIF-1α, β-catenin will rapidly switch roles from co-activating TCF-4 to stimulating HIF-1α mediated transcription [6]. An increase in HIF-1α mediated transcription under ischemic conditions leads to downstream VEGF upregulation with a range of possible sequelae including macular edema and/or ocular bleeding [6]. The present study indicates that emodin has an ameliorating effect on the ischemic damage done to the retinas, while at the same time there is a dose-dependent and significant (at 20 μM) downregulation of ischemia induced β-catenin/VEGF overexpression (Fig. 5). Therefore, the protective mechanisms of emodin would seem to involve a decrease in β-catenin protein levels (Fig. 5) together with a decrease in the aforementioned β-catenin co-activated HIF-1α mediated transcription [21] and a consequent reduction in VEGF concentrations (Fig. 5). This is consistent with previous publications [23–25] wherein it has been shown that emodin has anti-VEGF properties. In terms of clinical practicability, emodin seems to attenuate the ischemia associated elevation of VEGF levels, which are widely accepted to possibly lead to ocular hemorrhage (increased vascular permeability) and macular edema (fluid leak). All these findings and the present protein analysis (Fig. 5) strongly support the hypothesis that emodin has an anti-ischemic and protective effect probably via an inhibition of the ischemia induced overexpression of β-catenin and VEGF (Fig. 5). This is of major clinical importance, because it might point the way of managing clinically proved complications (e.g. macular edema) associated with the aforementioned retinal ischemic disorders. These include retinal vascular occlusion, proliferative diabetic retinopathy, neovascular age related maculopathy and*,* possibly, retinal developmental diseases such as Coats’ disease [1–3, 12, 26].

## Conclusion

The present study has demonstrated that raised IOP induced retinal ischemic injury is able to be alleviated by pre-ischemic and/or post-ischemic emodin application. The alterations induced by ischemia were monitored using electroretinography, histopathology (cresyl violet stained retinal layer thickness), retrograde fluorogold immunolabled RGCs, and measurement of protein expression levels of β-catenin and VEGF. In the MTT cell viability assay, pre-OGD administration of 0.5 μM emodin (an equivalent intravitreous injection dose of 20 μM in a rat) significantly attenuated the OGD induced cellular injury. Furthermore, in the animal study, pre-ischemic intravitreous injection of 20 μM emodin significantly attenuated the retinal ischaemia induced reduction in ERG b-wave amplitude. The post-ischemic intravitreous injection group also significantly alleviated this b-wave reduction. This protective effect was also present when cresyl violet stained retinal thickness and/or retrograde fluorogold immunolabeled RGC density were used. Additionally, β-catenin/VEGF protein levels have been significantly increased after retinal ischemic injury. These elevations were significantly blunted by pre-treatment with emodin. The above findings imply that emodin has a protective effect against retinal ischemia injured neurons such as RGCs via the downregulation of ischemia induced β-catenin/VEGF protein upregulation.

## Supplementary Information

**Additional file 1: Supplementary Material 1**. Animal Agreement

**Additional file 2: Supplementary Material 2**. Animal number

**Additional file 3: Supplementary Material 3**. ARRIVE Guidelines

**Additional file 4: Supplementary Material 4**. Emodin blot

## Data Availability

On reasonable request, data used and/or analyzed from the current study can be available from the corresponding author.

## Abbreviations

ERG: Electroretinogram
OCT: Optical coherence tomography
RGC: Retinal ganglion cells
IOP: Intraocular pressure
TCF-4: T-cell factor-4
HIF-1α: Hypoxia-inducible factor-1α
OGD: Oxygen glucose deprivation
MTT: 3-(4,5-dimethylthiazol-2-yl)-2,5-diphenyltetrazolium bromide
IR: Ischemia plus reperfusion
Emo: Emodin
ARRIVE: Animal Research: Reporting of In Vivo Experiments
ILM: Internal limiting membrane
RPE: Retinal pigment epithelium
INL: Inner nuclear layer
SDS-PAGE: Sodium dodecyl sulfate polyacrylamide gel electrophoresis
PVDF: Polyvinylidene fluoride
P: Probability
VEGF: Vascular endothelium factor
