# Supplementary figures and images for: Emodin protected against retinal ischemia insulted neurons through the downregulation of protein overexpression of β-catenin and vascular endothelium factor
Source: BMC Complement Med Ther. 2020 Nov 10;20:338. doi: 10.1186/s12906-020-03136-7 (PMC7654144; doi:10.1186/s12906-020-03136-7)

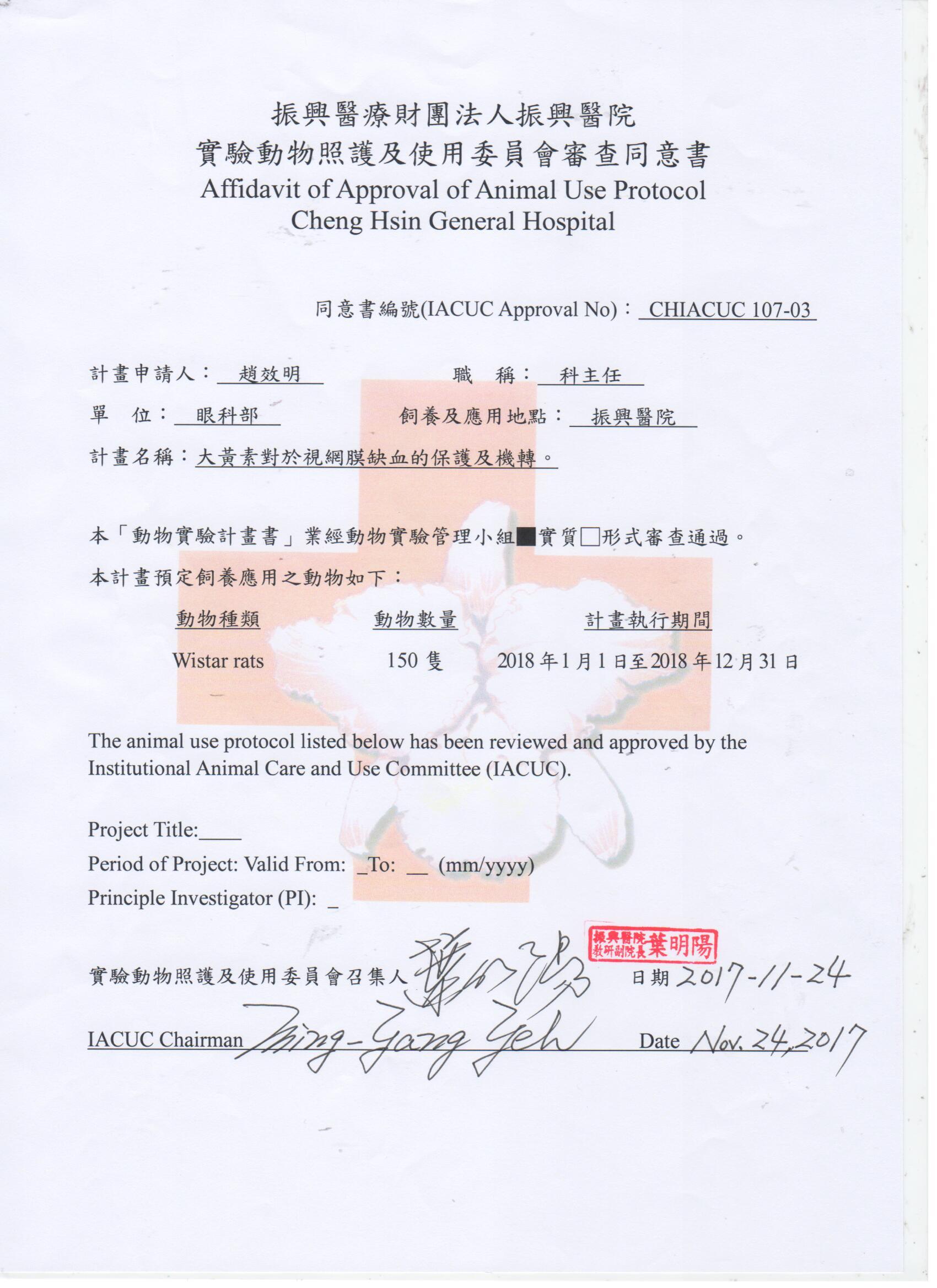

Supplement: Supplementary file 1 — Additional file 1: Supplementary Material 1. Animal Agreement [file 12906_2020_3136_MOESM1_ESM.jpg]
