# Supplementary material for: Emodin protected against retinal ischemia insulted neurons through the downregulation of protein overexpression of β-catenin and vascular endothelium factor
Source: BMC Complement Med Ther. 2020 Nov 10;20:338. doi: 10.1186/s12906-020-03136-7 (PMC7654144; doi:10.1186/s12906-020-03136-7)
Supplement: Supplementary file 2 — Additional file 2: Supplementary Material 2. Animal number [file 12906_2020_3136_MOESM2_ESM.docx]

Group/Method CV† FG† WB† Total*

Sham 10 4 5 19

Vehicle+IR 10 4 6 20

Emo4+IR 10 – – 10

Emo10+IR 10 4 6 20

Emo20+IR 10 4 6 20

IR+Emo20 10 – – 10

Subtotal No. 60 16 23 99

The number of Wistar rats initially scheduled to be utilised in this project was 150 (n=150). Due to this project grant being initially insufficiently afforded, the schedule for the real-time polymerase chain reaction procedure (n=30) was eventually discontinued. *The number of the rats used in this study is 120 (=99+21; 99=60+16+23), including animals (n=21) that died during the following procedures, namely in the HIOP (n=9), ERG (n=4), and FG (n=8). †After ERG recordings, all recorded rats (n=99) were preserved for the following procedures, namely CV, FG, and WB. Abbreviations: Sham, normal control; I/R, ischemia/reperfusion; Veh, vehicle; Emo4, Emodin 4 μM; Emo10, Emodin 10 μM; Emo20, Emodin 20 μM; CV, Cresyl violet stain; FG, fluorogold; WB, western blot; ERG, electroretinogram.
