# Supplementary material for: Emodin protected against retinal ischemia insulted neurons through the downregulation of protein overexpression of β-catenin and vascular endothelium factor
Source: BMC Complement Med Ther. 2020 Nov 10;20:338. doi: 10.1186/s12906-020-03136-7 (PMC7654144; doi:10.1186/s12906-020-03136-7)

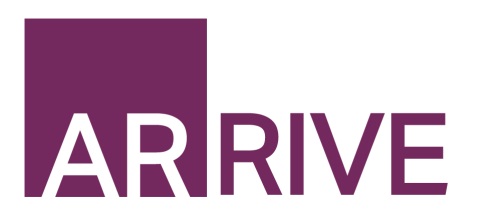


The ARRIVE Guidelines Checklist

Animal Research: Reporting In Vivo Experiments

Carol Kilkenny^1^, William J Browne^2^, Innes C Cuthill^3^, Michael Emerson^4^ and Douglas G Altman^5^

*^1^The National Centre for the Replacement, Refinement and Reduction of Animals in Research, London, UK, ^2^School of Veterinary Science, University of Bristol, Bristol, UK, ^3^School of Biological Sciences, University of Bristol, Bristol, UK, ^4^National Heart and Lung Institute, Imperial College London, UK, ^5^Centre for Statistics in Medicine, University of Oxford, Oxford, UK.*

|  | | ITEM | RECOMMENDATION | Section/ Paragraph |
| --- | --- | --- | --- | --- |
| 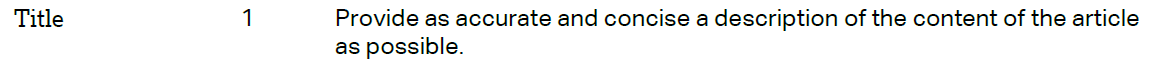 | | | Title |  |
| 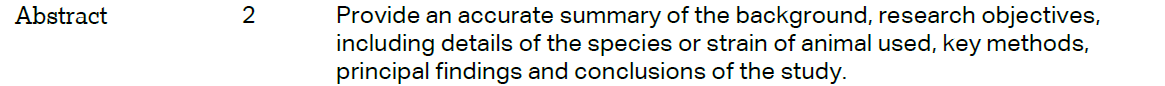 | | | Abstract |  |
| INTRODUCTION | | |  |  |
| 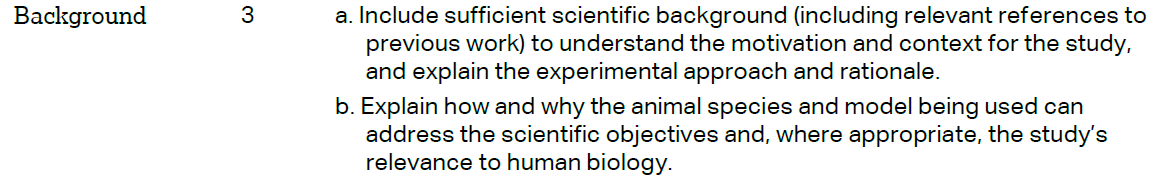 | | | **a.** Background **(Last paragraph).**  **b.** Background **(Paragraph 1)** & **Methods (HIOP).** The traditional Chinese Medicine Emodin was used to evaluate its protective effects & mechanisms against retinal ischemia. Experimental retinal ischemia (HIOP) was induced in a Wistar rat to mimic the human retinal ischemia. |  |
| 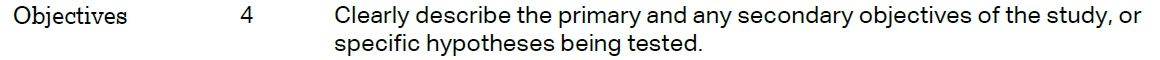 | | | Background (**The rationale of administration of emodin in retinal ischemia animal model**) |  |
| METHODS | | |  |  |
| 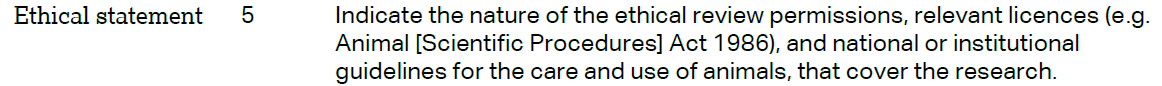 | | | Supplementary Material 1 (Animal Experiment Protocol & Agreement)  and  **Material and methods:** animal use  **Materials and methods:** animal anaesthesia and euthanasia  The Animal Use Approval has been agreed by the Institutional Animal Care and Use Committee at Cheng Hsin General Hospital, Taipei, Taiwan (Approval No: CHIACUC 107–03; Supplementary file 1). |  |
| 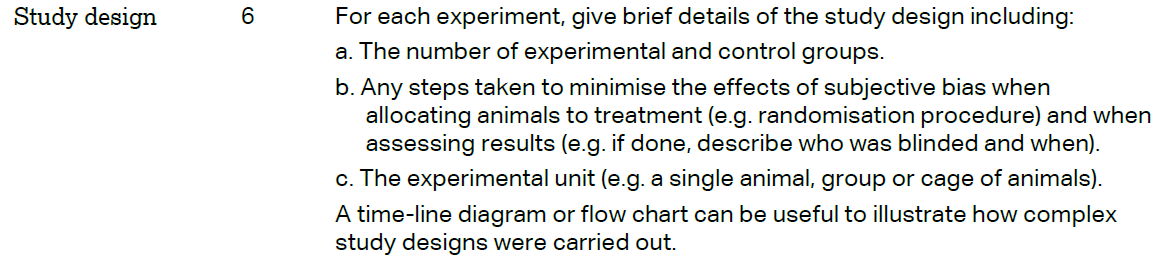 | | | Supplementary Material 2  See respective headers in Methods.   1. **Animal use** 2. **Animal anesthesia and euthanasia** 3. **Administration of drug; ERG recording; Cresyl violet stain; Retrograde labelling of RGCs; Western Blotting** |  |
| 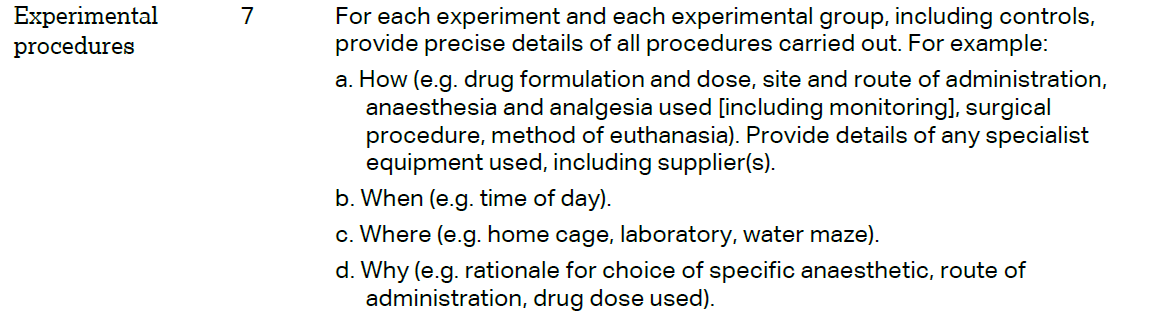 | | | Supplementary Material 1  Supplementary Material 2   1. Administration of drug; ERG recording; Cresyl violet stain; Retrograde labelling of RGCs; Western Blotting 2. Administration of drug 3. Animal use 4. **Background:** The rationale of administration of emodin in retinal ischemia animal model |  |
| 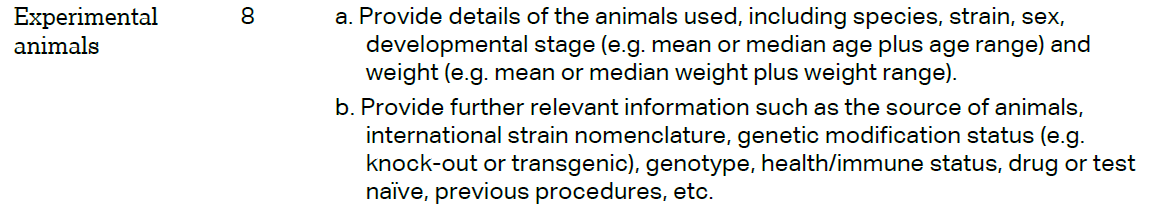 | | | Supplementary Material 1 (Animal Experiment Protocol & Agreement)  a & b. Materials and methods: Animal use |  |

The ARRIVE guidelines. Originally published in *PLoS Biology*, June 2010^1^

| 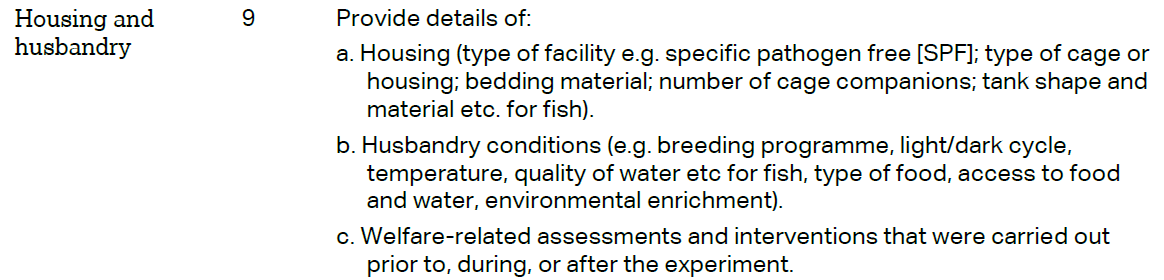 | a, b, c:  **Materials and methods:** Animal use |  |
| --- | --- | --- |
| 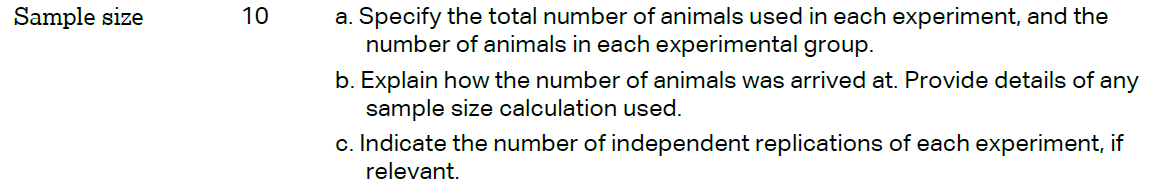 | a, b, c:  Supplementary material 1.  Supplementary material 2.  Animals [Totally, 120 rats (n = 120) were used]. Sham (n = 19), Vehicle + IR (n = 20), Emo4+IR (n = 10), Emo10+IR (n = 20), Emo20+IR (n = 20), IR+Emo20 (n=10). Twenty-one animals died during the following procedures, namely in the HIOP (n=9), ERG (n=4), and FG (n=8). |  |
| 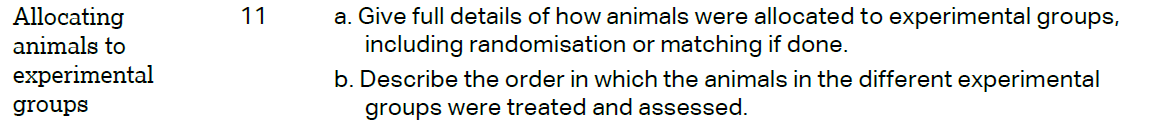 | a. **Materials and methods:** Animal use  b. Materials and methods: Animal use |  |
| 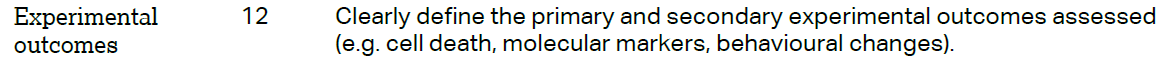 | Introduction (The rationale of administration of emodin in retinal ischemia animal model)  Materials and methods: Administration of drug; ERG recording; Cresyl violet stain; Retrograde labelling of RGCs; Western Blotting |  |
| 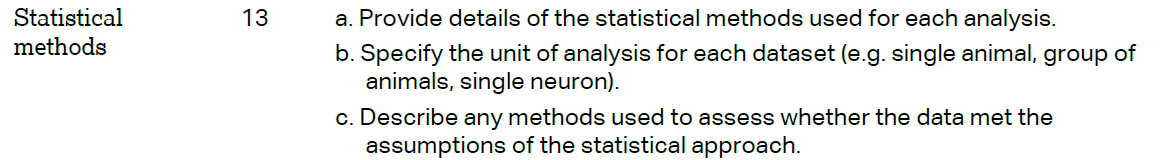 | **Materials and methods:** Analysis of statistical significance |  |
| RESULTS |  |  |
| 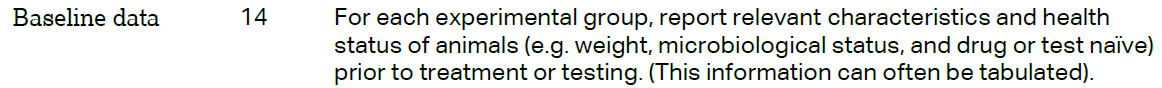 | See Methods (Animals & other sections using electroretinographic / histopathological / biochemical tests, e.g. Sham ERG b ratio ≈ 1 / FG RGC ≈ 5323 number/0.17mm^2^/ Cresyl violet outer retina ≈ 187 μm / Western blot β-catenin/VEGF Sham protein ≈ 1 ) |  |
| 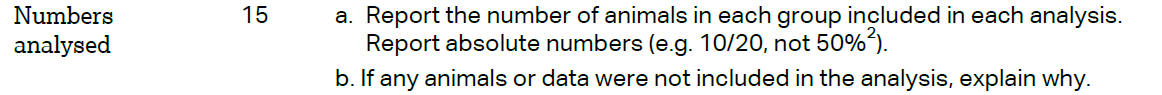 | a. See “Results” various sections & “Arrive Guide” ITEM 10 (Sample size)  b. No, none was not included |  |
| 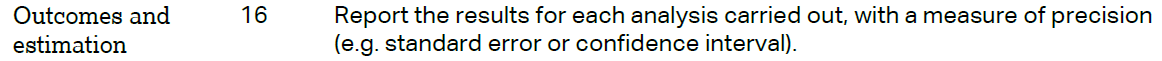 | **mean±SE (Statistical analysis, last sentence)** |  |
| 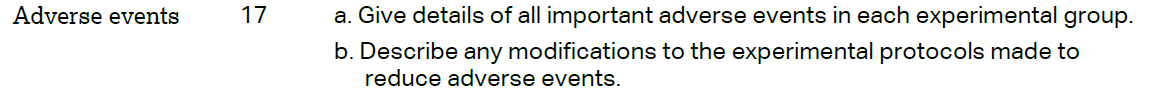 | **a & b. Surgical pain that can be reduced by anesthetics.** Supplementary Material 1 **(Animal Experiment Protocol & Agreement) & Methods (Animal anesthesia and euthanasia)** |  |
| DISCUSSION |  |  |
| 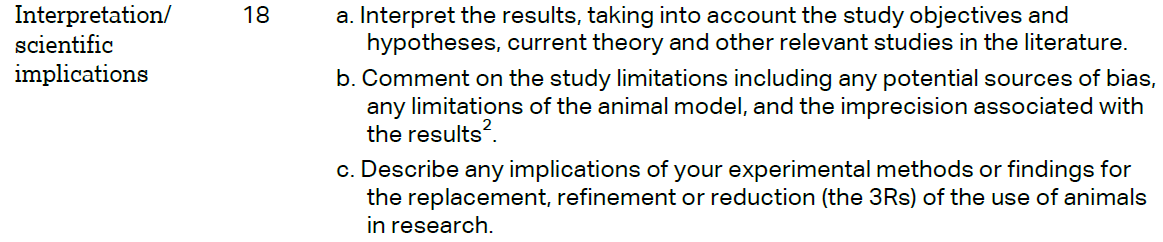 | **a. Paragraphs 2&3**  **b. Experimental ischemia mimics human retinal ischemia related ocular disorders, namely retinal vascular occlusion, PDR, AMD, retinal developmental diseases, e.g. Coats’ disease. These disorders, though all ischemic ones, are substantially different.**  c. Supplementary Material 1 **(Animal Experiment Protocol & Agreement) & “Arrive Guide” ITEM 10 (Sample size)**  **c. Supplementary material 2** |  |
| 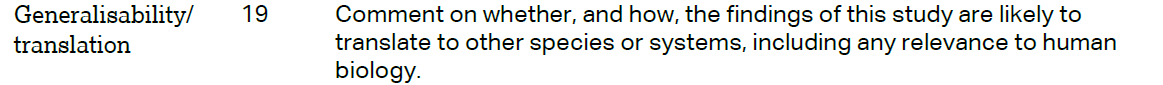 | Introduction: clinical relevance to the retinal ischaemic model |  |
| 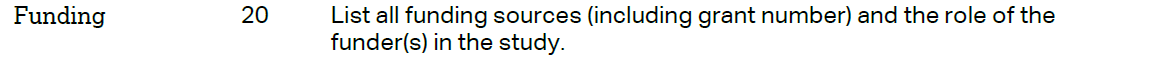 | | See acknowledgement & Bibliography  Grant number: 107-17 |


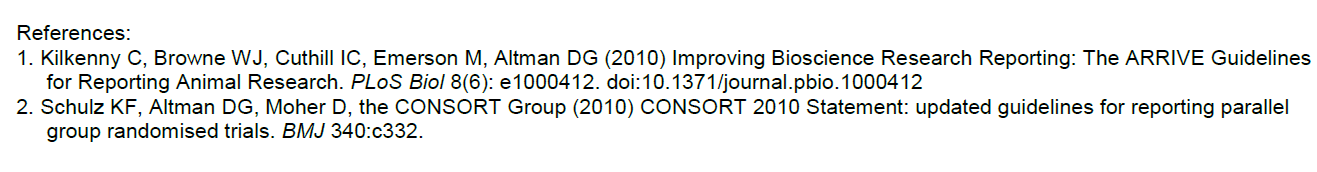

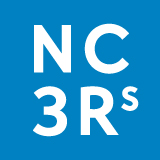

Supplement: Supplementary file 3 — Additional file 3: Supplementary Material 3. ARRIVE Guidelines [file 12906_2020_3136_MOESM3_ESM.docx]
