# Supplementary material for: Emodin protected against retinal ischemia insulted neurons through the downregulation of protein overexpression of β-catenin and vascular endothelium factor
Source: BMC Complement Med Ther. 2020 Nov 10;20:338. doi: 10.1186/s12906-020-03136-7 (PMC7654144; doi:10.1186/s12906-020-03136-7)
Supplement: Supplementary file 4 — Additional file 4: Supplementary Material 4. Emodin blot [file 12906_2020_3136_MOESM4_ESM.pdf]

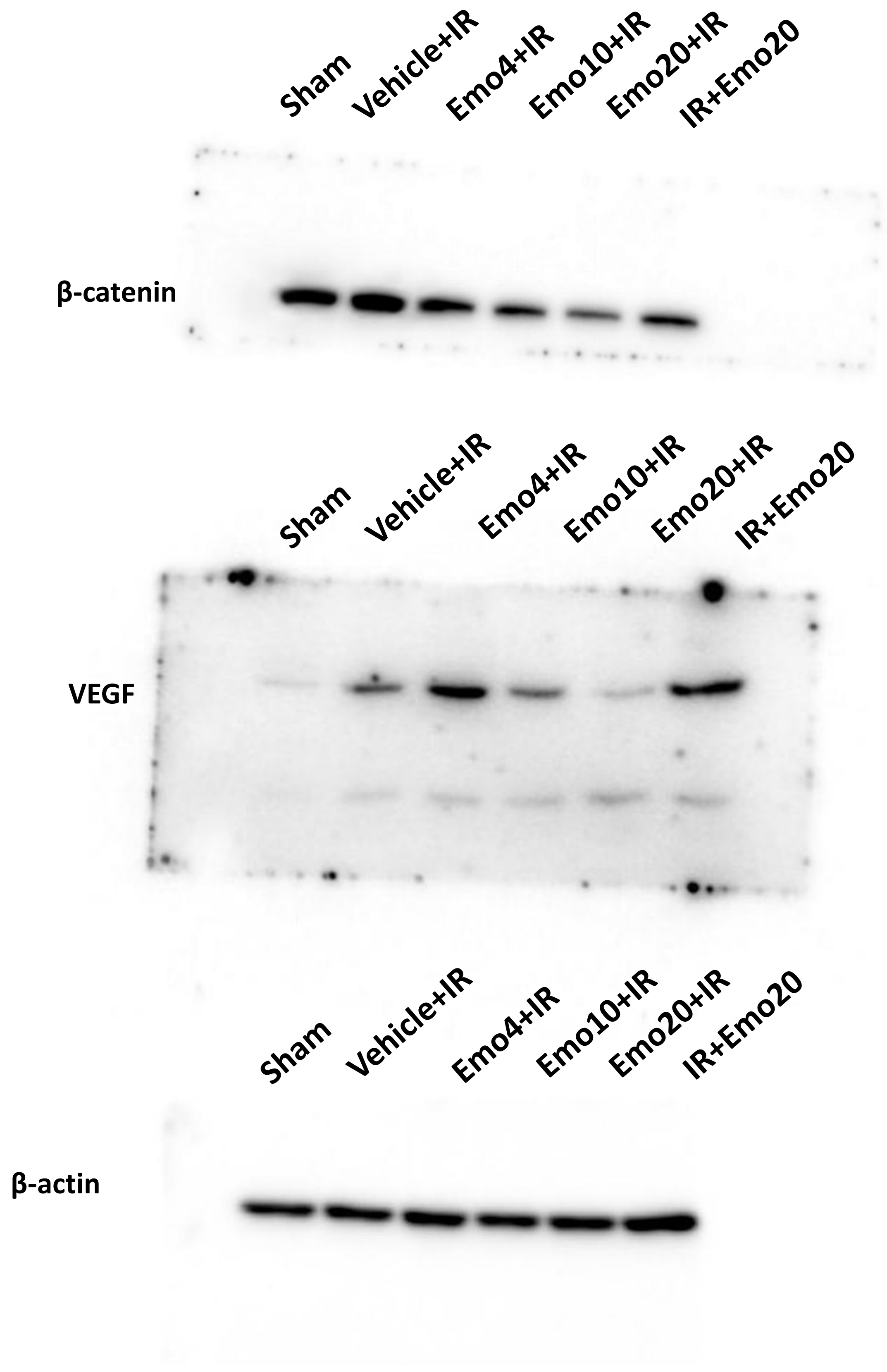

Supplementary Material 4. Western blot assay. The representative blotting images of β-catenin/vascular endothelium growth factor (VEGF)/β-actin. Lane 1 is from a sham retina (Control); Lane 2 is the vehicle-pretreated ischemic retina (Vehicle+IR); Lanes 3, 4, 5 and 6 are from retinas that were subjected to IR with pre-ischemic treatment with 4/10/20 μM emodin (Emo4+IR/Emo10+IR/Emo20+IR) or post-ischemic administration of 20 μM emodin (IR+Emo20). As compared to the normal control, the VEGF/β-catenin protein level obviously increased after retinal ischemia with pre-ischemic intravitreous vehicle (Vehicle+IR) was carried out. During the first trial using western blotting assay, this VEGF/β-catenin elevation in the ischemic retina was not/not obviously counteracted by pre-ischemic administration of 4 μM emodin or post-ischemic treatment with 20 μM emodin. For the clarity and conciseness of information these two groups were cropped out of the figure in the manuscript. In the following experiments, pre-ischemic administration of 10 and 20 μM concentrations of emodin were thus selected/tested to find out the dose-dependent effects on the ischemic retina. As demonstrated, the elevated levels of VEGF/β-catenin in the ischemic retina were in a dose-response manner downregulated by pre-ischemic treatment with 10 (with a less effect) or 20 μM emodin (with a greater effect).
